# Disordered eating and the meat-avoidance spectrum: a systematic review and clinical implications

**DOI:** 10.1007/s40519-022-01428-0

**Published:** 2022-06-21

**Authors:** Courtney P. McLean, Jayashri Kulkarni, Gemma Sharp

**Affiliations:** Monash Alfred Psychiatry research centre, Monash University, 4/607 St Kilda Road, Melbourne, VIC 3004 Australia

**Keywords:** Veganism, Vegetarianism, Eating disorders, Orthorexia nervosa, Psychometric properties

## Abstract

**Purpose:**

Meat avoidance has long been thought to be related to eating psychopathology; however, research does not necessarily support this notion. Furthermore, commonly used eating disorder scales may be picking up on normal meat-avoiding behaviours in vegetarians and vegans. As such, we systematically reviewed the association between vegetarianism, veganism, and disordered eating, and reviewed the psychometric properties of eating disorder scales for use in these populations.

**Methods:**

We searched electronic databases MEDLINE, PsychINFO, and CINAHL for literature published until June 2021.

**Results:**

Forty-eight studies met eligibility criteria, with no consensus as to whether meat avoidance was associated with higher rates of disordered eating. Most studies reported a significant positive association with both vegetarianism and veganism, and orthorexia nervosa. Six studies provided evidence for the use of eating disorder measures in vegetarians and vegans, reporting poor psychometric fit among all scales.

**Conclusion:**

This systematic review highlights the extent to which vegetarians and vegans have been highly understudied, with limited research suggesting higher levels of orthorexia nervosa behaviours in vegetarians and vegans. Furthermore, our results provide tentative evidence that the factorial validity of commonly used eating disorder scales, such as the EDE-Q, may be poor in vegans.

**Level of evidence:**

Level I, systematic review.

It has long been thought that vegetarianism and veganism are related to an elevated risk of disordered eating; however, past research does not necessarily support this notion [1, 2]. Vegetarianism, defined as a dietary pattern involving the exclusion of red meat, and often poultry, fish, and seafood, is considered diverse and heterogenous in nature, encapsulating a number of wide-ranging dietary variations (i.e., lacto-vegetarianism, semi–vegetarianism) [3]. Additional restrictions are imposed on those following a vegan diet (i.e., the exclusion of all animal-derived products), with many incorporating lifestyle modifications such as rejecting clothing or entertainment involving exploitation of, or cruelty to, animals [4]. According to Rosenfeld and Burrow’s [5] dietary identity theory, vegetarians and vegans consider their diet to be a central part of their identity, display more positive feelings about their dietary in-group, and feel more negatively judged for following their dietary pattern relative to omnivores [6, 7]. These findings suggest that meat avoidance is considered by some to be more than just a diet, but rather an interlacing of one’s identity when it comes to food.

The idea that veg*ism, used herein to indicate the spectrum of meat avoidance, is associated with greater rates of disordered eating has not been well established. At its very core, veg*ism involves a high level of cognitive restraint to consciously regulate and restrict several food groups. While dietary motivations for veg*ism do not appear to influence disordered eating rates [8–11], it has been posited that veg*ism in and of itself may act as a socially acceptable method to restrict food intake and camouflage disordered eating behaviours [12–16]. Orthorexia nervosa, a form of disordered eating characterised by a pervasive obsession to eat “clean” and “pure” foods, has shown the strongest link with veg*ism due to similar overlapping food selection strategies [17]. For example, both orthorexia nervosa and veg*ism allow individuals to reduce their food intake according to specific nutritional rules (e.g., consumption of low sugar or gluten-free diet, abstaining from meat products) resulting in a diet of very few foods. While both facilitate efforts at dietary restriction, veg*ism may allow its followers to legitimize this food avoidance, potentially enabling greater disordered eating behaviours [16].

It is possible that some eating disorder scales may be capturing normal veg*an-motivated food choices and behaviours such as higher levels of cognitive restraint due to a heavy avoidance of certain food groups [1]. This could result in inaccurate estimates of the prevalence of eating disorders in these populations. Some examples of potentially confusing items in commonly used eating disorder scales to veg*ans are displayed in Table 1. For example, the Eating Disorder Examination-Questionnaire (EDE-Q) asks respondents to rate the degree they exclude foods and use food rules to influence weight or shape which may rely on insight that their dietary restrictions are indeed weight or shape motivated to be accurate. A limited number of studies have assessed the psychometric properties of commonly used eating disorder scales in veg*ans, producing largely inconsistent results. This demonstrates a significant gap within the literature, which is particularly important considering the utility of these tools (e.g., EDE-Q) as screening instruments and outcome measures.

**Table 1:** Examples of potentially confusing items in eating disorder scales for veg*ans

EAT-40 [18]	Item 2. Prepare foods for others but do not eat what I cook -Veg*ans may frequently prepare foods with animal products for others Item 19. Enjoy eating meat -Veg*ns do not eat meat and may be biased in their response Item 30. Eat diet foods -Veg*n foods are often considered to be “diet” foods Item 32. Display self-control around food -Veg*ans must display self-control around foods containing animal products Item 33. Feel that others pressure me to eat -Veg*ans may feel pressure to eat animal products by family and friends
EDE-Q [19]	Item 3 (Restraint Subscale). Have you tried to exclude from your diet foods that you like in order to influence your shape or weight? -Veg*ans routinely exclude animal products as part of their diet. This item also relies on the respondent being aware and honest about their motivations for these exclusions Item 4 (Restraint Subscale). Have you tried to follow definite rules regarding your eating in order to influence your shape or weight? -Veg*ans routinely follow definite rules around the exclusion of animal products as part of their diet. This item also relies on the respondent being aware and honest about their motivations for following rules
ORTO-15 [20]	Item 2. When you go to a food shop do you feel confused? -Veg*ans may often feel confused when reading ingredients lists to assess whether they contain animal products Item 8. Do you allow yourself any eating transgressions? -Veg*ans do not allow eating transgressions within the realm of animal products
TFEQ [21]	Item 1 (Cognitive Restraint Subscale). When I smell a sizzling steak or see a juicy piece of meat, I find it very difficult to keep from eating, even if I have just finished a meal -Veg*ans do not eat meat and may be biased in their response. We encourage the use of Forestell, Spaeth and Kane [22]’s modified item (“When I smell a chocolate cake baking or see a delicious cookie, I find it very difficult to keep from eating, even if I have just finished a meal”) when administering the TFEQ to veg*ans
YFAS [23]	Item 11 (Important social, occupational, or recreational activities given up or reduced Subscale). There have been times when I avoided professional or social situations because I was not able to consume certain foods there -Veg*ans may avoid professional or social situations where there is limited veg*an options Item 23. I have tried to cut down to stop eating certain kinds of food -Veg*ans regularly restrict their diet to ensure they do not consume animal products Item 24 (Persistent desire or repeated unsuccessful attempts to quit Subscale). I have been successful at cutting down or not eating these kinds of foods -Veg*ans regularly restrict their diet to ensure they do not consume animal products

*EAT*  eating attitudes test, *EDE*-*Q*  eating disorder examination-questionnaire, *TFEQ* three-factor eating questionnaire, *YFAS*  Yale food addiction scale

The aim of this study is to conduct a systematic review of all published studies to ascertain the association between vegetarianism, veganism, and disordered eating. To our knowledge, only one systematic review has investigated the relationship between vegetarian diets and disordered eating. Sergentanis [24] collated the results of 20 studies finding a positive relationship between veg*ism and disordered eating in adolescents and young adults. Our systematic review represents an addition to Sergentanis [24]’s important work by investigating disordered eating along the meat-avoidance spectrum in a wider age range (i.e., adults aged 16 years and over included). Furthermore, we extend our systematic review to a secondary aim to examine the psychometric properties of eating disorder scales for use in vegetarians and vegans. Finally, we will then discuss the potential clinical implications of employing commonly used eating disorder scales to diagnose and monitor treatment progress in veg*ans with a suspected eating disorder.

## Methods

This systematic review was reported in accordance with the Preferred Reporting Items for Systematic Reviews and Meta-Analyses (PRISMA) statement [25]. A priori registration to PROSPERO was received on 4 May 2021 (registration number CRD42021244365). A manual literature search was conducted in June 2021 in electronic bibliographic databases MEDLINE, PsychINFO, and CINAHL. A combination of search or MeSH terms, appearing in either title, abstract, subject heading, or keyword, were used, including vegan* OR vegetarian* OR “plant based” OR “meat avoid*” OR “meat abstain*”, with “eating disorder*” OR “disordered eating*” OR anorexi* OR bulimi* OR orthorexi* OR “binge eat*”. The reference lists of eligible papers were searched for relevant studies reporting on psychometric properties of eating disorder scales in veg*ans for inclusion.

### Inclusion criteria

Inclusion criteria were based on the PICOS format (Participant, Intervention, Comparison, Outcomes, Study Design) [26], whereby articles eligible for inclusion in this review were required to meet the following criteria.

#### Types of participants

Studies that reported on individuals 16 years and over were eligible. This age range was chosen because we elected to focus on independent individual food choices rather than family-based food choices.

#### Types of interventions

Studies on individuals who follow a vegetarian (i.e., excluding red meat) or vegan (i.e., excluding animal-derived food products including meat, poultry, fish, eggs, and milk) diet were eligible.

#### Types of comparisons

Studies that used omnivores (i.e., consuming animal and plant products) as the reference group were eligible.

#### Types of outcomes

Studies assessing the relationship between vegetarianism, veganism, and disordered eating/eating disorders were eligible. We also elected to include studies assessing the relationship between vegetarianism, veganism, and orthorexia nervosa due to its characterisation is a form of disordered eating with overlapping symptoms as a subtype of anorexia nervosa [27].

#### Types of studies

Case studies, letters, conference abstracts or posters, systematic reviews, meta-analyses, extant reviews, and narrative reviews were restricted for inclusion. Grey literature dissertations were eligible. No publication date restrictions were imposed.

### Exclusion criteria

Animal studies and studies that reported on samples under the age of 16 were excluded. Studies were restricted to those written in English language.

### Study selection

First author independently screened studies against eligibility criteria using systematic review management platform, Covidence. Studies were screened in a hierarchical fashion, whereby titles and abstracts of searched literature were screened first, followed by full text to identify studies that met inclusion criteria. Eligible papers were imported and stored in database management software, Endnote, to allow for easy data extraction. If multiple articles for a single study were available, the most up-to-date publication was used.

### Data extraction and synthesis

Tables were used to synthesise study characteristics and results. Study characteristics and results include first author name, date of publication, country of origin, study design, sample size, vegetarian and vegan sample size, veg*an age, veg*an gender, veg*an ethnicity/race, veg*an socioeconomic status, vegetarian and vegan study definition, study population, eating disorder measure, main findings including effect size of the correlation between veg*ism and disordered eating if available, and Newcastle–Ottawa scale quality rating. Due to large heterogeneity between study samples (e.g., *N* = 45 to 10,137), diet type (e.g., vegetarianism, semi–vegetarianism, laco-vegetarianism, veganism), and measures (e.g., orthorexia nervosa, restraint, disordered eating, binge eating, food addiction) [28], it was not possible to pool data into a statistical meta-analysis. Therefore, a quantitative synthesis is provided.

### Quality assessment

To assess extraction bias, review author, GS blindly reviewed 10% of full-text articles to ensure inclusion and exclusion accuracy. This involved review author being unaware of the inclusion status of the manuscript during the assessment. If discrepancies did arise during the bias assessment, disagreements were discussed and resolved by consensus, or through the inclusion of third author.

To assess methodological quality, all studies were assessed using a modified Newcastle–Ottawa scale for cross-sectional [29], retrospective, or case–control studies [30]. The Newcastle–Ottawa scale allocates points to each study evaluating three domains: selection, comparability, and outcome/exposure. Cross-sectional studies were allocated a maximum of ten points, with higher scores consistent with greater methodological rigor (high = 9–10, good = 7–8, satisfactory = 5–6, poor = 0–4). Retrospective and case–control studies were allocated a maximum of 9 points, with an overall rating of high (> 6), moderate (4–5), or poor (0–3), with high ratings indicative of greater methodological rigor. Review author, GS blindly assessed 10% of studies. If discrepancies did arise during the quality assessment, disagreements were discussed and resolved by consensus, or through the inclusion of third author.

## Results

### Study selection

**Fig. 1:**
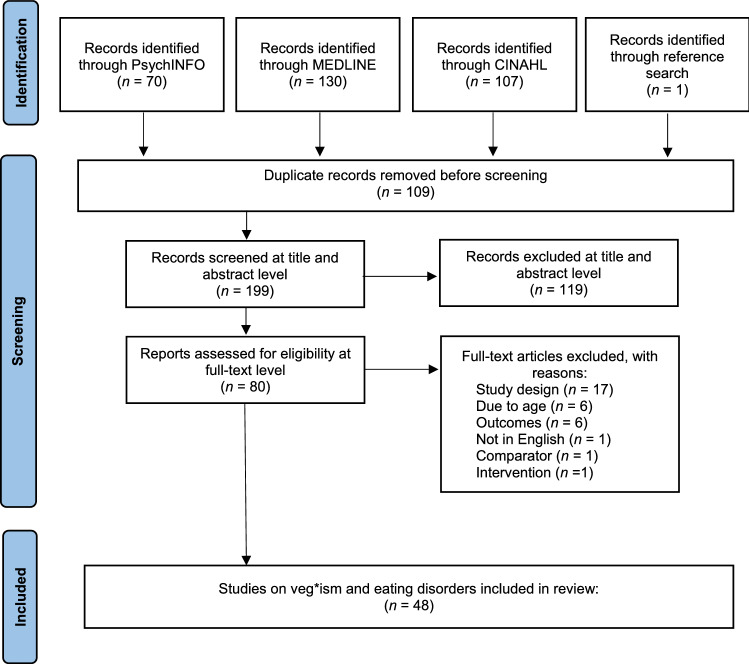
PRISMA flow diagram of study selection process

The initial literature search identified a total of 308 potentially relevant studies. After removal of duplicates, 199 were screened at title and abstract level with 119 deemed not relevant. A further 80 studies were evaluated for eligibility at full-text level with 32 excluded due to not meeting selection criteria. A discrepancy of four papers was identified during review author’s extraction check and were resolved through discussion and subsequently included in the review. Ultimately, a total of 48 studies were included in the systematic review. A PRISMA flow diagram describing the successive steps of the selection process is presented in Fig. 1.

### Quality assessment of included studies

The methodological quality of most cross-sectional studies was satisfactory, followed by poor quality. The main limitation across studies was no description of the response rate or characteristics between respondents and non–respondents. A major strength across studies was the utilisation of valid and reliable measurement tools, such as the EDE-Q and Eating Attitudes Test (EAT). Of the three studies of retrospective design, in terms of overall rating, one study received a moderate-quality rating, and two received a poor-quality rating. The one case–control study was rated as high quality. The main limitation across retrospective and case–control studies was unclear follow-up rates or description of participants lost. A main strength across the studies was the representativeness of the veg*an samples. The quality ratings of the 48 studies are presented in Table 2.

**Table 2:** Characteristics of included studies

Author (Year)	Country	Study Design	Total Sample Size	Vegetarian Sample Size	Vegan Sample Size	Veg*an Age Distribution	Veg*an Gender Distribution (Female)	Veg*an Ethnicity/ Race (White)	Veg*an Socio-Economic Status	Definition of Vegetarianism	Definition of Veganism	Study Population	Measure of Interest	Global Disordered Eating Findings and Effect Sizes (if available)	Orthorexia Nervosa Findings and Effect Sizes (if available)	NOS
Bardone-Cone [16]	USA	Cross-sectional	160	26	NR	NR	100.0%	NR	NR	Excluding beef	NR	Eating disorder patients and community sample	DSM-IV	History of eating disorder was associated with greater likelihood of having ever being vegetarian and currently vegetarian compared to no eating disorder history	–	7/10
Barrack [31]	USA	Cross-sectional	106	5	NR	18.0	80.0%	NR	NR	NR	NR	University students	EDE-Q	Vegetarianism was associated with greater disordered eating	–	5/10
Barthels [17]	Germany	Cross-sectional	351	63	114	Vegetarian = 30.7 (10.1), Vegan = 28.7 (8.6)	Vegetarian = 72.6% Female, Vegan = 71.6% Female	NR	NR	Excluding red meat	Excluding red meat, fish, poultry, and rarely consuming dairy and eggs	Community sample	DOS, RES	No association between diet and greater restraint	Vegetarianism and veganism were associated with greater orthorexia behaviours than rare and frequent meat eating (*p* < .001, *ƞ*^*2*^ = 0.13)	7/10
Barthels, Poerschke, Mueller and Pietrowsky [55]	Germany	Cross-sectional	65	0	65	28.2 (9.1)	53.9%	NR	NR	NR	Excluding meat, fish, poultry, dairy, eggs, and gelatine	Community sample	DOS	–	The vegan sample fell within the 70th percentile of the normal population	5/10
Bas [32]	Turkey	Cross-sectional	1,205	29*	2	Female = 20.6 (1.8), Male = 21.5 (1.3)	74.2%	NR	NR	NR	NR	University students	EAT-26	Vegetarianism was associated with greater disordered eating	–	5/10
Brytek-Matera [50]	Poland	Cross-sectional	370	188	NR	28.9 (10.3)	NR	NR	NR	Excluding meat	NR	Community sample	TFEQ-R18-Polish, EHQ	Vegetarianism was associated with lower disordered eating, measured by cognitive restraint (*p* < .001, *d* = 0.55)	Vegetarianism was associated with greater orthorexia behaviours, healthy eating (*p* < .001, *d* = 0.98), problems with healthy eating (*p* < .001, *d* = 0.78), and positive feelings to healthy eating (*p* < .001, *d* = 0.67)	6/10
Brytek-Matera [49]	Poland	Cross-sectional	254	100	47	Vegetarian = 28.4 (8.9), Vegan = 30.6 (11.6)	NR	NR	NR	Excluding all animal-derived foods such as	Excluding all animal products such as dairy,	Community sample	TFEQ-Polish, EHQ-Polish	Vegetarianism and veganism were associated with lower disordered eating,	Vegetarianism and veganism were associated with greater orthorexia behaviours,	5/10
Author (Year)	Country	Study Design	Total Sample Size	Vegetarian Sample Size	Vegan Sample Size	Veg*an Age Distribution	Veg*an Gender Distribution (Female)	Veg*an Ethnicity/ Race (White)	Veg*an Socio-Economic Status	Definition of Vegetarianism	Definition of Veganism	Study Population	Measure of Interest	Global Disordered Eating Findings and Effect Sizes (if available)	Orthorexia Nervosa Findings and Effect Sizes (if available)	NOS
										meat, poultry, and fish	cheese, and eggs			measured by cognitive restraint (*p* < .001, *ƞ*^*2*^ = 0.089), emotional eating (*p* < .001, *ƞ*^*2*^ = 0.107), and uncontrolled eating (*p* < .001, *ƞ*^*2*^ = 0.184)	measured by healthy eating (*p* < .001, *ƞ*^*2*^ = 0.59), knowledge with healthy eating (*p* < .001, *ƞ*^*2*^ = 0.223), and positive feelings to healthy eating (*p* < .01, *ƞ*^*2*^ = 0.61)	
Brytek-Matera [42]	Poland	Cross-sectional	120	39	40	Vegetarian = 26.5 (8.1), Vegan = 29.7 (10.8)	NR	NR	NR	Excluding meat	Excluding all animal products	Community sample	TFEQ-R18-Polish, EHQ-Polish	No association between diet and greater disordered eating, measured by cognitive restraint (*p* > .05, *ƞ*^*2*^ = 0.027), emotional eating (*p* > .05, *ƞ*^*2*^ = 0.006), and uncontrolled eating (*p* > .05, *ƞ*^*2*^ = 0.026)	Vegetarianism and veganism were associated with greater orthorexia behaviours, measured by healthy eating (*p* < .001, *ƞ*^*2*^ = 0.165), knowledge with healthy eating (*p* < .001, *ƞ*^*2*^ = 0.249), and positive feelings to healthy eating (*p* < .01, *ƞ*^*2*^ = 0.099)	6/10
Çiçekoğlu and Tunçay [43]	Turkey	Cross-sectional	62	16*	15	32.7 (5.6)	71.0%	NR	Omnivores were 6.5% more likely to hold a tertiary degree or higher	NR	NR	Community sample	ORTO-11, EAT-40	No association between diet and greater disordered eating	No association between diet and greater orthorexia behaviours	5/10
Collins and Quinton [33]	Australia	Cross-sectional	634	104	281	Vegetarian = 28.9 (11.3), Vegan = 27.5 (10.1)	100.0%	NR	NR	Excluding red meat, poultry, and fish	Excluding all animal-derived products	University and community sample	EAT-26	Semi–vegetarianism was associated with greater disordered eating, followed by non–vegetarians, vegetarians, and vegans	–	5/10
Dell’Osso [56]	Italy	Cross-sectional	2130	240*	NR	NR	75.0%	NR	NR	NR	NR	University students	ORTO-15	–	Vegetarianism/ veganism was associated with greater orthorexia behaviours	5/10
Dell'Osso [57]	Italy	Cross-sectional	2826	313*	NR	76.7% < 29 years, 23.3% > 29 years	73.8%	NR	Parent education level did not significantly differ between diet	NR	NR	University student and staff sample	ORTO-15	–	Vegetarianism/veganism was associated with greater orthorexia behaviours	5/10
Author (Year)	Country	Study Design	Total Sample Size	Vegetarian Sample Size	Vegan Sample Size	Veg*an Age Distribution	Veg*an Gender Distribution (Female)	Veg*an Ethnicity/ Race (White)	Veg*an Socio-Economic Status	Definition of Vegetarianism	Definition of Veganism	Study Population	Measure of Interest	Global Disordered Eating Findings and Effect Sizes (if available)	Orthorexia Nervosa Findings and Effect Sizes (if available)	NOS
Delucca [44]	USA	Cross-sectional	70	33	19	Vegetarian = 60.6% 20–29, Vegan = 52.6% 20–29	Vegetarian = 81.8%, Vegan = 68.4%	Vegetarian = 87.9%, Vegan = 89.5%	NR	Excluding all types of meat	Excluding meat, dairy, egg, or any other products derived from animals	Community sample	EAT-26	No association between diet and greater disordered eating (*p* > .05, *ƞ*^*2*^*p* = 0.02)	–	4/10
Dittfeld [58]	Poland	Cross-sectional	2,611	1,346	NR	Vegetarian = 25.6 (8.7), Vegan = 25.7 (8.4)	Vegetarian = 89.9%, Vegan = 87.2	NR	Vegetarians were significantly more likely to hold a higher education	Excluding meat, fish, and seafood	Excluding all forms of animal products	NR	BOT	–	Lacto–vegetarianism was associated with greater orthorexia behaviours, followed by ovo–vegetarians, lacto–ovo–vegetarians, and vegans	4/10
Dorard and Mathieu [45]	France	Cross-sectional	101	49*	NR	28.6 (11.6)	85.7%	NR	Omnivores had significantly higher education levels than vegetarians	NR	NR	Community sample	EAT-26, BSQ	No association between diet and greater disordered eating (*p* = .219, *d* = –0.24). Vegetarianism was associated with lower pathological body concerns (*p* = .043, *d* = –0.41)	–	7/10
Dunn, Gibbs, Whitney and Starosta [59]	USA	Cross-sectional	275	28	6	NR	NR	NR	NR	NR	NR	University students	ORTO-15	–	No association between diet and orthorexia nervosa (*p* = .79, *ƞ*^*2*^ = 0.04). Veganism was associated with lower orthorexia behaviours when compared with those endorsing no restrictions (*p* = .01, *ƞ*^*2*^ = 0.34)	5/10
Ferreira and Coimbra [60]	Portugal	Cross-sectional	541^b^	66	60	NR	NR	NR	NR	NR	NR	Community sample	DOS	–	Veganism was associated with greater orthorexia behaviours, followed by vegetarians and omnivores	4/10
Fisak [46]	USA	Cross-sectional	256	48*	4	NR	100.0%	NR	NR	Excluding meat	Excluding meat, egg, or dairy products	University students	EDI-II, DEBQ, EAT, TFEQ,	No association between diet and greater disordered eating	–	4/10
Author (Year)	Country	Study Design	Total Sample Size	Vegetarian Sample Size	Vegan Sample Size	Veg*an Age Distribution	Veg*an Gender Distribution (Female)	Veg*an Ethnicity/ Race (White)	Veg*an Socio-Economic Status	Definition of Vegetarianism	Definition of Veganism	Study Population	Measure of Interest	Global Disordered Eating Findings and Effect Sizes (if available)	Orthorexia Nervosa Findings and Effect Sizes (if available)	NOS
Forestell [22]	USA	Cross-sectional	240	41*	16	Vegetarian = 19.4 (0.2)	100.0%	NR	Family income (% > $75,00) did not differ between diets	NR	NR	University students	TFEQ, EAT	No association between diet and greater disordered eating	–	4/10
Gilbody [34]	United Kingdom	Cross-sectional	131	44*	1	Vegetarian = 20.0	100.0%	NR	NR	Excluding meat and fish	Excluding animal products	University students	DEBQ	Vegetarianism was associated with greater dietary restraint. No association between diet and emotional eating and external eating	–	3/10
Heiss [54]	USA	Cross-sectional	518	0	318	31.8 (12.6)	82.3%	90.9%	NR	NR	Abstaining from all animal products	University and community sample	EDE-Q	Veganism was associated with lower restraint (*p* < .001, *ƞ*^*2*^*p* = 0.16), but not associated with greater eating (*p* = .77, *ƞ*^*2*^*p* < 0.001), shape, (*p* = .67, *ƞ*^*2*^*p* < 0.001), or weight concerns (*p* = .53, *ƞ*^*2*^*p* < 0.01)	–	7/10
Heiss [10]	USA	Cross-sectional	577	0	245	31.3 (12.4)	83.7%	88.5%	NR	NR	Refraining from all animal products	Community sample	EDE-Q, DEBQ, EDI, BES, YFAS	Veganism was associated with lower disordered eating measured by EDE–Q (*p* < .001, *ƞ*^*2*^*p* = 0.05). No association between diet and greater disordered eating measured by the EDI (*p* = .05, *ƞ*^*2*^*p* = 0.01), BES (*p* = .19, *ƞ*^*2*^*p* < 0.01), DEBQ, and YFAS (*p* = .13, *ƞ*^*2*^*p* = 0.01)	–	9/10
Heiss, Timko and Hormes [61]^a^	USA	Cross-sectional	518	0	318	31.8 (12.6)	82.3%	90.9%	NR	NR	Refraining from all animal products	University and community sample	EDE-Q	–	–	4/10
Heiss [51]	USA	Cross-sectional	381	50	191	Vegetarian = 27.8 (9.8), Vegan = 31.7 (12.9)	Vegetarian = 84.0%, Vegan = 82.2%	Vegetarian = 88.0%, Vegan = 90.6%	NR	Refraining from all animal flesh	Refraining from all animal products	Community sample	EDE-Q, ORTO-15	Veganism was associated with lower disordered eating, followed by vegetarians, omnivores, and meat reducers (*p* = .001, *ƞ*^*2*^*p* = 0.04)	Veganism was associated with greater orthorexia behaviours, followed by meat reducers, vegetarians, and omnivores (*p* < .001, *ƞ*^*2*^*p* = 0.06)	8/10
Author (Year)	Country	Study Design	Total Sample Size	Vegetarian Sample Size	Vegan Sample Size	Veg*an Age Distribution	Veg*an Gender Distribution (Female)	Veg*an Ethnicity/ Race (White)	Veg*an Socio-Economic Status	Definition of Vegetarianism	Definition of Veganism	Study Population	Measure of Interest	Global Disordered Eating Findings and Effect Sizes (if available)	Orthorexia Nervosa Findings and Effect Sizes (if available)	NOS
Heiss [2]	USA	Retrospective chart design	124	20	5	Vegetarian = 22.8 (9.9), Vegan = 20.0 (5.3)	Vegetarian = 95.0%, Vegan = 60.0%	Vegetarian = 100.0%, Vegan = 80.0%	NR	NR	NR	Eating disorder patients	EAT-26, MAEDS	No association between diet and greater disordered eating (*p* = .52, *ƞ*^*2*^*p* = 0.02)	–	4/9
Herranz Valera, Acuna Ruiz, Romero Valdespino and Visioli [62]	Spain	Cross-sectional	136	38	7	NR	Vegetarian = 55.3%, Vegan = 42.9%	NR	NR	NR	NR	Ashtanga yoga practitioners	ORTO-15	–	Vegetarianism was associated with greater orthorexia behaviours. No association between veganism and greater orthorexia behaviours	3/10
Hessler-Kaufmann [63]	Germany	Cross-sectional	511	49*	NR	33.8 (13.0)	77.6%	NR	NR	Excluding meat, poultry, and fish	Excluding all products derived from animals	Community sample	DOS	–	Vegetarianism was associated with greater orthorexia behaviours, followed by semi–vegetarians, and omnivores	6/10
Janelle and Barr [52]	Canada	Cross-sectional	45	15*	8	Vegetarian = 25.8 (4.7), Vegan = 28.0 (3.2)	100%	NR	NR	Excluding meat, fish, and poultry	Excluding meat, fish, poultry, and dairy products	NR	TFEQ	Vegetarianism was associated with lower restraint, but not associated with greater hunger and disinhibition	–	4/10
Kadambari [35]	United Kingdom	Retrospective study	180	77	NR	NR	NR	NR	There was no significant association between diet and lower social class	NR	NR	Eating disorder patients	Intensity of current “weight phobia”, feeding patterns	Vegetarianism was associated with greater intensity of avoidance of “fatness” and feeding abstinence. No association between diet and feeding patterns of bulimia, vomiting, purging, hunger, or inability to eat in the presence of others	–	1/9
Klopp [12]	USA	Cross-sectional	143	30	0	19.0 (1.0)	100%	NR	NR	NR	Consuming no animal origin foods	University students	EAT-40	Vegetarianism was associated with greater disordered eating	–	5/10
Lacey and Zotter [47]	USA	Cross-sectional	92	8	0	NR	100%	NR	NR	NR	NR	University students	EAT-26	No association between diet and greater disordered eating	–	5/10
Author (Year)	Country	Study Design	Total Sample Size	Vegetarian Sample Size	Vegan Sample Size	Veg*an Age Distribution	Veg*an Gender Distribution (Female)	Veg*an Ethnicity/ Race (White)	Veg*an Socio-Economic Status	Definition of Vegetarianism	Definition of Veganism	Study Population	Measure of Interest	Global Disordered Eating Findings and Effect Sizes (if available)	Orthorexia Nervosa Findings and Effect Sizes (if available)	NOS
Lindeman [14]	Finland	Cross-sectional	124^b^	14	NR	NR	100%	NR	NR	Excluding red and white meat and fish	NR	University and community sample	EAT, EDI	Vegetarianism was associated with greater disordered eating	–	4/10
Luck-Sikorski, Jung, Schlosser and Riedel-Heller [64]	Germany	Cross-sectional	1,007	NR	NR	NR	NR	NR	NR	NR	NR	Community sample	DOS	–	Vegetarianism was associated with greater orthorexia behaviours	5/10
McLean and Barr [36]	Canada	Cross-sectional	596	47	NR	NR	100%	NR	NR	Excluding meat, fish, and poultry	NR	University students	TFEQ-15	Vegetarianism was associated with greater restraint	–	5/10
Micali [37]	United Kingdom	Cross-sectional	10,137	NR	NR	NR	100%	NR	NR	A negative loading for red meat and poultry	NR	Women in their third trimester of pregnancy	-	Women with eating disorders were more likely to describe themselves as vegetarian compared to women with no reported eating disorder (OR 2.8, 95% CI 2.1, 3.8)	–	6/10
Missbach [65]	Austria	Cross-sectional	1,029	NR	NR	NR	NR	NR	NR	NR	NR	Community sample	ORTO-15	–	Vegetarianism was associated with greater orthorexia behaviours, followed by vegans, and mixed diet	5/10
Norwood [53]	Australia	Cross-sectional	393	48	128	Vegetarian = 27.4, Vegan = 32.5	Vegetarian = 88.0%, Vegan = 83.0%	Vegetarian = 80.0%, Vegan = 84.0%	NR	NR	NR	University and community sample	EDI-5, DEBQ-13, DIS-7	Vegetarianism and veganism were associated with lower disordered eating	–	8/10
O'Connor [48]	Australia	Retrospective study	116	63	0	NR	NR	NR	NR	The avoidance of meat	Avoiding the consumption of all animal products	Eating disorder patients	Weight loss behaviours	No association between diet and greater weight loss behaviours	–	3/9
Oberle, De Nadai and Madrid [66]	USA	Cross-sectional	847	42	78	NR	NR	NR	NR	Excluding red meat, poultry, and fish	Excluding red meat, poultry, fish, eggs, dairy, or any animal by-products	University and community sample	ONI	–	Veganism was associated with greater orthorexia behaviours, followed by vegetarians and semi–vegetarians, when compared to non–vegetarians	5/10
Author (Year)	Country	Study Design	Total Sample Size	Vegetarian Sample Size	Vegan Sample Size	Veg*an Age Distribution	Veg*an Gender Distribution (Female)	Veg*an Ethnicity/ Race (White)	Veg*an Socio-Economic Status	Definition of Vegetarianism	Definition of Veganism	Study Population	Measure of Interest	Global Disordered Eating Findings and Effect Sizes (if available)	Orthorexia Nervosa Findings and Effect Sizes (if available)	NOS
Parra-Fernández [67]	Spain	Cross-sectional	466	109	101	Vegetarian = 29.2 (11.1), Vegan = 33.3 (11.1)	Vegetarian = 82.6%, Vegan = 78.2%	NR	NR	NR	NR	Community sample	ORTO-11-ES	–	Veganism was associated with a higher risk for orthorexia nervosa, followed by vegetarians and omnivores	5/10
Paslakis [38]	Canada	Cross-sectional	2,449	133*	NR	40.9 (15.5)	73.7%	NR	Vegetarians had significantly higher education levels than omnivores	Omitting meat but eating plants and milk products	Omitting all foods of animal origin	Community sample	EDE-Q8	Vegetarianism was associated with greater disordered eating	–	5/10
Robinson-O'Brien [39]	USA	Cross-sectional	1,692^c^	76	NR	NR	76.5%	NR	NR	NR	NR	Community sample	Binge eating question, Weight-control behaviours questions	No association between diet and healthful weight control behaviours and less extreme weight-control behaviours. Vegetarianism was associated with greater more-extreme unhealthful weight-control behaviours, and binge eating	–	7/10
Sieke [8]	USA	Cross-sectional	1,585	128*	8	NR	NR	NR	NR	Excluding red meat	NR	University students	EDE-Q	Semi–vegetarianism was associated with greater disordered eating, followed by vegetarians, and omnivores (*p* < .001, *d* = 0.22)	–	5/10
Timko [9]	USA	Cross-sectional	486	111	35	Vegetarian = 26.7 (9.1), Vegan = 26.9 (7.9)	Vegetarian = 86.0%, Vegan = 86.0%	NR	NR	NR	Excluding all animal products	University and community sample	DEBQ, EAT-26, Drive for Thinness (DT) subscale of EDI-3	No association between diet and EAT–26 and DEBQ emotional eating (*p* = .12, *ƞ*^*2*^*p* = 0.01). Semi–vegetarianism was associated with higher DEBQ restraint, followed by vegetarians, omnivores, and vegans (*p* < .01, *ƞ*^*2*^*p* = 0.03). Veganism	–	5/10
Author (Year)	Country	Study Design	Total Sample Size	Vegetarian Sample Size	Vegan Sample Size	Veg*an Age Distribution	Veg*an Gender Distribution (Female)	Veg*an Ethnicity/ Race (White)	Veg*an Socio-Economic Status	Definition of Vegetarianism	Definition of Veganism	Study Population	Measure of Interest	Global Disordered Eating Findings and Effect Sizes (if available)	Orthorexia Nervosa Findings and Effect Sizes (if available)	NOS
														was associated with lower DEBQ external eating, followed by vegetarians, semi–vegetarians, and omnivores (*p* < .001, *ƞ*^*2*^*p* = 0.04). Veganism was associated with greater drive for thinness, followed by omnivores, vegetarians, and semi–vegetarians (*p* = .02, *ƞ*^*2*^*p* = 0.02)		
Trautmann [13]	USA	Cross-sectional	330	30	0	NR	93.3%	NR	NR	Excluding red meat	Excluding all animal products	University students	DEBQ, EAT-26	Vegetarianism was associated with greater disordered eating	–	4/10
Yackobovitch-Gavan [40]	Israel	Case control design	90	NR	NR	NR	NR	NR	NR	NR	NR	University students and eating disorder patients	EDFHI, DSM-IV	Vegetarianism was associated with greater prevalence of non–remission (1/OR 10.58, 95% CI 0.011, 0.789)	–	7/9
Zickgraf [41]	USA	Cross-sectional	9,910	822*	146	NR	81.0%	NR	NR	NR	NR	University students	S-EDE-Q	Weight-motivated vegetarianism was associated with greater disordered eating relative to omnivores and non–weight-motivated vegetarians (*p* < .001, *ƞ*^*2*^*p* = 0.013)	–	7/10
Zuromski [15]	USA	Cross-sectional	278	NR	NR	NR	100%	NR	NR	Regularly eating dairy and egg products, but not meats	Excluding animal products	University students and eating disorder patients	-	History of vegetarianism was associated with greater likelihood of a diagnosed eating disorder, followed by a subclinical diagnosis, and no lifetime eating pathology	–	5/10

*NR* not reported, *DSM* diagnostic statistical manual, *EDE*-*Q* eating disorder examination-questionnaire, *DOS* Dusseldorf orthorexia scale, *RES* restraint eating scale, *EAT* eating attitudes test, *TFEQ* three-factor eating questionnaire, *TFEQ*-*R* TFEQ-restraint, *EHQ* eating habits questionnaire, *BOT* Bratman test for orthorexia, *BSQ* body shape questionnaire, *EDI* eating disorder inventory, *DEBQ* Dutch eating behaviour questionnaire, *BES* binge eating scale, *YFAS* Yale food addiction scale, *MAEDS* multifactorial assessment of eating disorders symptoms, *FFQ* food frequency questionnaire, *DIS* dieting intentions scale, *ONI* orthorexia nervosa inventory, *EDFHI* eating disorders family history interview, *NOS* Newcastle–Ottawa scale

*Denotes combined vegetarian and vegan sample for analysis

^a^Study found through reference list search providing psychometric results in veg*ans—no association between disordered eating to report

^b^Results presented for sample 2

^c^Results presented for older cohort

### Study characteristics

The study characteristics of the 48 included studies are presented in Table 2. Most studies (*n* = 35) were published within the last 10 years and primarily based in the U.S. (*n* = 20). Forty-four of the 48 studies used a cross-sectional design, with the remaining four studies using retrospective and case–control design. The total sample size of studies ranged from *N* = 45 to *N* = 10,137.

### Relationship between veg*ism and disordered eating

#### Study characteristics

Thirty-six studies were identified that reported on the relationship between veg*ism and global disordered eating. Veg*an sample size of studies ranged from *N* = 5 to *N* = 822. Nineteen studies provided criteria for defining vegetarianism in their sample which ranged from excluding beef to all forms of animal flesh, and 17 providing criteria for defining veganism which ranged from excluding meat, egg, or dairy products to all animal-derived products. Mean veg*an sample age ranged from 19.0 (*SD* = 1.0) years in the youngest sample to 40.9 (*SD* = 15.5) years in the oldest sample. Most studies used mix-gender samples (*n* = 17), with 12 studies using female-only samples. No studies reported an even gender distribution among the veg*an group, with females significantly outweighing males in all studies. Six studies reported veg*an ethnicity/race, supporting predominantly White/Caucasian participants. Five studies reported veg*an SES. Community samples and university students were the most common study population (both *n* = 11), followed by mixed university student and community samples (*n* = 6), eating disorder patients (*n* = 3), university students and eating disorder patients (*n* = 2), mixed eating disorder patient and community samples (*n* = 1), and women in their third trimester of pregnancy (*n* = 1). The most common disordered eating measure was the EAT (*n* = 13), followed by the EDE-Q (*n* = 8) and Dutch Eating Behaviour Questionnaire (DEBQ; *n* = 6).

#### Vegetarian samples

A range of statistical tests showed a significant positive association between vegetarianism and disordered eating in eighteen of 33 studies [8, 9, 12–16, 31–41]. Sixteen studies reported no association between vegetarianism and disordered eating [2, 9, 17, 22, 31, 34, 35, 39, 42–48], and seven studies reported a negative association between vegetarianism and disordered eating [9, 45, 49–53]. Of note, some studies reported differing associations depending on the specific eating disorder measure used. For example, Dorard and Mathieu [45] found higher disordered eating when measured by the Body Shape Questionnaire (BSQ), but not when measured by the EAT-26. There is also little consensus within eating disorder measures. For example, when measured using the EAT, five studies reported greater disordered eating in the vegetarian sample than omnivores, compared to eight studies that reported no association between vegetarianism and disordered eating.

#### Vegan samples

Most studies (8 of 13) reported no association between veganism and disordered eating [2, 9, 10, 17, 42–44, 54]. Six studies reported lower disordered eating in the vegan sample [9, 10, 33, 49, 51, 53], with two studies reporting greater disordered eating in the vegan sample compared to omnivore controls [9, 15]. Like the vegetarian results, findings of studies differed depending on the eating disorder measure or subscale used. For example, Heiss [10] reported lower disordered eating in vegans when measured by the EDE-Q, but no association when measured by the Eating Disorder Inventory (EDI), Binge Eating Scale (BES), Yale Food Addiction Scale (YFAS), and DEBQ. Similarly, Timko [9] reported no association in veg*ans when measured by the EAT-26 and DEBQ emotional eating subscale, but a negative association between veganism and the DEBQ dietary restraint and emotional eating subscales.

### Relationship between veg*ism and orthorexia nervosa

#### Study characteristics

Eighteen studies were identified that reported on the relationship between veg*ism and orthorexia nervosa, a form of disordered eating characterised by a pervasive obsession to eat “clean” and “pure” foods [17]. The sample size of studies ranged from *N* = 62 to *N* = 2,826, with veg*an sample sizes ranging from *N* = 6 to *N* = 1,346. Of the 17 studies with vegetarian participants, eight provided criteria for defining vegetarianism in their sample. Of the 14 studies with vegan participants, eight provided criteria for defining veganism in their sample. Veg*an sample age was predominately based in the mid to late 20s. In line with Relationship between Veg*ism and Disordered Eating, gender distribution was heavily dominated by females. Information on veg*an ethnicity/race were rarely provided, with one study reporting a predominately White/Caucasian sample (88.0–90.6%). Three studies reported veg*an SES. The most common measure to assess orthorexia nervosa was the ORTO (*n* = 8), followed by the Dusseldorf Orthorexia Scale (DOS; *n* = 5), and Eating Habits Questionnaire (EHQ; *n* = 3).

#### Vegetarian samples

A range of statistical tests showed a significant positive association between vegetarian adherence and orthorexia nervosa pathology (15 of 16) [17, 42, 49, 50, 56–58, 60, 62–66]. One study reported no association between vegetarianism and orthorexia nervosa pathology [43], and no studies reported a negative association between vegetarianism and orthorexia nervosa pathology.

#### Vegan samples

Nine of 12 studies reported a significant positive association between veganism and orthorexia nervosa pathology [17, 42, 49, 51, 55, 58, 60, 66, 67]. One study reported a negative association with orthorexia nervosa pathology [59], while two studies reported no association between veganism and orthorexia nervosa pathology compared to omnivore controls [43, 62]. Notably, those that reported a negative or no association between veganism and orthorexia had substantially smaller veg*an sample sizes. For example, Dunn [59] reported veganism was associated with lower orthorexia nervosa pathology measured by the ORTO-15 in a sample of six vegans (equivalent to 2% of the total sample size). Similarly, Herranz Valera [62] reported no association between veganism and orthorexia nervosa pathology using a sample of seven vegans.

### Psychometric properties of eating disorder scales in veg*ans

To address the secondary aim of this systematic review, the psychometric properties of eating disorder scales applied in veg*an samples were reviewed and synthesized (see Table 3). Of the 48 studies included in this review, six studies provided evidence for the use of eating disorder scales in veg*ans. Most studies assessed the EDE-Q (*n* = 5), followed by the DEBQ and EDI (*n* = 2). One study examined an orthorexia nervosa measure, the ORTO-15. Notably, four studies are reported by the same author, with two studies using the same sample [54, 61].

**Table 3:** Summary of the psychometric properties of eating disorder measures in veg*ans

Author (Year)	Measure	Veg*an sample	Psychometric properties
Fisak [46]	EAT, EDI, DEBQ, TFEQ	Vegetarians	Reliability Cronbach’s alpha a = .91 for EAT, .91 for EDI-DT, .87 for EDI-B, .95 for DEBQ, .92 for TFEQ-CR, .78 for TFEQ-D, and .83 for TFEQ-H
Heiss [10]	EDE-Q, DEBQ, EDI-DT, DM, BES, YFAS	Vegans	Reliability Cronbach’s alpha ranged from .80 to .90 for the EDE-Q subscales, .75 to .96 for the DEBQ subscales, .88 for EDI-DT, .88 for the DM, .86 for BES, and .92 for YFAS
Heiss [54]	EDE-Q	Vegans	Reliability Cronbach’s alpha ranged from .81 to .94 on all subscales of the four-, three, two-, and full one-, and brief one-factor scales Composite reliability ranged from 0.82 to 0.94 on all subscales of the four-, three-, two-, full one-, and brief one-factor scales Confirmatory Factor Analysis^a^ Model fit was unacceptable in the four-, three-, two-, and full one-, and brief one-factor models. Heywood case was observed in the four-factor model in both samples
Heiss [51]	ORTO-15, EDE-Q	Vegetarians and vegans	Reliability Cronbach’s alpha for the EDE-Q ranged from .82 to .94 for the vegan group and .79 to .94 for the vegetarian group. Cronbach’s alpha for the ORTO-15 = .37 for the vegan group and a = .42 for the vegetarian group Item-total correlations were significant for all items, except item two, with correlation coefficients ranging from small to large Validity ORTO-15 scores were significantly negatively correlated with EDE-Q restraint scores in vegans, but not vegetarians. OTRO-15 scores were unrelated to EDE-Q global, eating, shape, and weight scores across all groups
Heiss [61]	EDE-Q	Vegans	Reliability Cronbach’s alpha ranged from .83 to .96 across subscales Confirmatory Factor Analysis^b^ Model fit was good for the brief three-factor model Validity Configural invariance was supported. A test of metric invariance found the factor loadings were non–equivalent across the vegan and omnivore groups
Zickgraf [41]	S-EDE-Q	Vegetarian/vegans	Reliability Cronbach’s alpha for the global and subscales were good Confirmatory Factor Analysis^c^ Model fit was adequate for the three-factor model in the full sample. Model fit of the three-factor model was not conducted on the vegetarian/vegan sample Validity Configural, metric, scalar, and residual measurement invariance was supported across non–vegetarians, weight-motivated vegetarians, and non–weight-motivated vegetarians

*EAT* eating attitudes test; *EDI*-*DT* eating disorder inventory-drive for thinness scale; *DM* drive for muscularity; *EDI*-*B* eating disorder inventory—bulimia scale; *DEBQ* Dutch eating behavior questionnaire; *TFEQ*-*CR* three-factor eating questionnaire—cognitive restraint scale; *TFEQ*-*D* three-factor eating questionnaire—disinhibition scale; *TFEQ*-*H* three-factor eating questionnaire—hunger scale; EDE-Q: eating disorder examination-questionnaire; *BES* binge eating scale; *YFAS* Yale food addiction scale

^a^Three-factor model = restraint, eating concern, shape/weight concern, two-factor model = restraint, eating/shape/weight concern, one-factor model = global, brief one-factor model = brief weight and shape concern (items 11, 22, 23, 24, 25, 26, 27, 28)

^b^Brief three-factor model = restraint, shape/weight over-evaluation, body dissatisfaction (items 1, 3, 4, 22, 23, 25, 26)

^c^Short-three-factor model = restraint, shape/weight over-evaluation, body dissatisfaction (items 1, 3, 4, 22, 23, 25, 26)

In terms of reliability, good to excellent internal consistency of the EDE-Q was reported in vegans [10, 51, 54, 61], and good internal consistency was reported in combined veg*ans [41]. Among the other reported measures, acceptable to good internal consistency was reported for the DEBQ and TFEQ, and good to excellent internal consistency was reported for the EDI, EAT, Drive for Muscularity, BES, and YFAS in vegetarians, vegans, and combined samples [10, 46]. Internal consistency for the ORTO was found to be unacceptable in both vegetarian and vegan groups [51].

In terms of model fit, three studies conducted confirmatory factor analysis (CFA) using the EDE-Q reporting contradictory results. Heiss [54] found difficulties in replicating model fit of the four-factor model in addition to the three-, two-, and full one- and brief one-factor models in a sample of vegans. Further research found adequate but slightly less support for the brief three-factor model than the omnivore group [61].

Measurement invariance was documented in two studies using the EDE-Q in samples of vegans and vegetarians. Heiss [61] reported the factor loadings of the EDE-Q were not equivalent between the vegan and omnivore group. However, Zickgraf [41] supported full measurement invariance between non–vegetarians, weight-motivated vegetarians, and non–weight-motivated vegetarians.

## Discussion

We present the first systematic review to examine the association between vegetarianism, veganism, and disordered eating in adults of all ages. This review is also the first to synthesize the psychometric properties of eating disorder scales for use in vegetarian and vegan populations. A total of 48 studies met eligibility criteria, with most studies being cross-sectional in nature. Samples comprised primarily community samples of young adult women of Caucasian descent. Importantly, this review highlights the extent to which vegetarians and vegans have been highly understudied in the eating disorder/disordered eating research field. As veg*ism is likely proving to be to be more than just another “passing fad” [68], future research is vital to untie the complex relationship between disordered eating and veg*an eating behaviours and attitudes.

Our review showed there is no consensus whether vegetarianism or veganism is associated with higher levels of disordered eating. Specifically, our review was unable to confirm whether diet type (e.g., semi–vegetarianism, veganism) influenced rates of disordered eating. For example, we found studies reporting higher disordered eating in vegans [e.g., 9, 51, 60, 66], vegetarians [e.g., 58, 63, 65], semi–vegetarians [e.g., 9, 33], meat reducers [e.g., 51], and omnivores [e.g., 9] compared to other subtypes among a range of scales. While restricting dietary groups based on animal products is expected to serve as a risk factor to developing an eating disorder, it may be the case that the number of dietary groups excluded (e.g., meat, diary) may not play as greater role [17]. Future research is required to disentangle the extent to which diet type serves as a risk factor to disordered eating habits, with longitudinal research required to confirm the causality of this association. Our review also showed that the relationship between vegetarianism, veganism, and disordered eating differed depending on the assessment measure employed [9, 10]. It is clear disordered eating is a broad construct, whereby different measures may be detecting slightly different aspects of disordered eating attitudes and behaviours. Future research should aim to examine the relationship between vegetarianism, veganism, and specific aspects of disordered eating, as this was seemingly the exception, rather than the norm in the studies reviewed.

While motivations for dietary adherence do not appear to influence disordered eating rates (i.e., being veg*an for health reasons vs. ideological/ethical reasons) [8–10, 69], lack of agreement discovered through this review may also be explained by additional factors, such as poor methodological quality. For example, smaller sample sizes of veg*an samples meant studies were likely statistically underpowered. Another possible explanation is a lack of consensus in defining subtypes of veg*ans. While vegetarians, vegans, and their respective subtypes have been shown to differ in meaningful ways, many studies continue to group them into one singular category, potentially masking true associations between each group. Stringency in ensuring distinct vegetarian and vegan samples is recruited will ensure greater confidence when drawing on results of future studies.

In contrast to the lack of agreement between veg*ism and disordered eating, vegetarianism and veganism appear to be associated with greater orthorexia nervosa pathology. Characterised by a fixation on eating “healthy” foods, such as through an increased concern about the health of ingredients, compulsive checking of ingredients lists, and cutting of food groups (e.g., sugar, gluten), orthorexia nervosa is not formally recognised in the Diagnostic and Statistical Manual of Mental Disorders (DMS) [70]. This lack of formalised diagnostic criteria is in part due to disagreement around how best to classify orthorexia, with some suggesting it shares common features with anorexia nervosa and obsessive compulsive disorder (OCD) [71]. Overlapping similarities between veg*ism and orthorexia nervosa have been characterised to include specific food selection according to nutritional rules and food-related issues becoming a large part of one’s day-to-day [42]. It remains unclear, however, as to whether orthorexia scores of those following a veg*an diet exceed cut-off for pathological orthorexia behaviours. Some found not only do vegetarians and vegans display greater orthorexia behaviours compared to omnivore controls, but they also reach preliminary cut-off for orthorexia nervosa, with vegans being more affected [17, 51].

The positive results of the systematic review must be considered with limitations of orthorexia nervosa scales in mind. For example, the most widely used scale, the ORTO, is broadly criticized for having inconsistent psychometric properties [72], and other scales, such as the DOS and EHQ, have been shown to detect both orthorexia nervosa and healthy orthorexia [73]. As veg*ism is associated with greater health consciousness (i.e., consume alcohol less frequently, exercise more often, have a higher daily intake of fruit and vegetables) [10, 39], it may be the case that veg*ans are high in healthy orthorexia, rather than orthorexia nervosa itself, but commonly used orthorexia tools are unable to disentangle these distinct constructs [74]. Future work must pay attention to measuring the multidimensional constructs of orthorexia nervosa to ensure we correctly account for psychopathology in populations of interest, including veg*ans. Ultimately, until validated and widely supported diagnostic criteria have been established, true orthorexia nervosa remains difficult to classify and the results of this systematic review must be considered in view of this.

Our systematic review found support for overall good psychometric properties of eating disorder scales (e.g., EAT, DEBQ) in veg*ans. These results should be considered in light of preliminary research that calls into question difficulties in replicating the factor structure of these tools. For example, our systematic review supported good reliability and validity of the EDE-Q but found limited support for an appropriate factor structure of the tool in vegetarians and vegans [75]. Given full measurement invariance of the EDE-Q was supported across samples of omnivores, weight-motivated vegetarians, and non–weight-motivated vegetarians, but not samples of vegans and omnivores [41], it may be the case that the EDE-Q is not measuring the same latent construct of disordered eating across vegetarian, vegan, and omnivore groups. Furthermore, the psychometric properties of orthorexia nervosa scale, the ORTO, was provided in one study revealing poor internal consistency and insufficient discriminant validity. Taken together with the findings that vegans had greater orthorexia nervosa scores, it may be the case that the ORTO is not detecting true pathological orthorexic eating behaviours but rather normal dietary adherence within this population. Taken together, this area presents a critical gap in eating disorder research, particularly considering these measures are being used to estimate eating disorder prevalence in the community, as well as support diagnostic options for vegetarians and vegans with a suspected eating disorder.

### Strengths and limits

There are several strength and limitations to this systematic review. This systematic review offers the most comprehensive examination of the association between vegetarianism, veganism, and disordered eating to date, and is the first to synthesise all available literature to inform the use of eating disorder scales in assessing veg*ans. Ultimately, this systematic review highlights that vegetarian and vegan research has been wholly understudied in the eating disorder research field. An important limitation is that due to a heavy reliance on cross-sectional studies, the causal relationship between veg*ism and disordered eating cannot be confirmed. It therefore remains unclear whether veg*ism increases the risk of developing disordered eating, or whether developing disordered eating increases the chances of transitioning to a vegetarian or vegan diet. Second, the significant amount of missing demographic information (e.g., gender, ethnicity/race) across studies raises concerns regarding the representativeness of the samples. From the available demographic information, there is also limited variability with participants comprising predominately young Caucasian women. While this is in line with vegan population estimates from existing literature [76, 77], generalisability of these findings is constrained. Furthermore, we defined vegetarianism as the exclusion of red meat compared to the exclusion of meat in general, resulting in an additional four known studies. This means we may have captured participants who are not strict vegetarians, such as pescatarians or semi–vegetarians, but does ensure inclusivity of the meat-avoidance spectrum. We also acknowledge that many studies did not provide a definition of veg*ism in their study, meaning that the number of combined vegetarian, semi–vegetarian, or pescatarian samples may be underestimated in our results. We encourage researchers to incorporate rigorous processes, such as the two-tier process to defining meat avoidance described in Asher [78], to assist in minimising ambiguity in definitions and creating a streamline approach to the research field. Finally, quality ratings within studies were overall low, indicating a high degree of risk of bias, likely characteristic of a heavy reliance on self-report data.

### Clinical implications

This systematic review has important clinical implications for veg*an populations. Although further clinical research is very much needed, it is possible that veg*ism, in and of itself, may not necessarily be a risk factor for the development of eating disorders. Furthermore, our results provide tentative evidence that the factorial validity of commonly used eating disorder scales, such as the EDE-Q, may be poor in veg*ans. According to our review, at this stage, the brief-three-factor model (also known as the short-three-factor model) of the EDE-Q provides the only promising lead in appropriately measuring eating disorder symptoms in veg*ans. Specifically, the brief-three-factor model contains items that query restraint to influence body weight or shape (i.e., *“Have you tried to exclude from your diet any foods that you like in order to influence your shape or weight?”, “Have you tried to follow definite rules regarding your eating in order to influence your shape or weight?”*) so as not to pick up on normal veg*an-related eating restraint. Though this may be troublesome itself, as it relies on a high level of insight from respondents that their dietary restrictions are indeed weight motivated to provide accurate responses. Furthermore, as the model contains seven items under three factors, it does not meet minimum recommendations for factor analysis (e.g., four items per latent variable), limiting reliability estimates and generalizability of the questionnaire [79]. Until eating disorder scales have undergone stringent psychometric testing in both vegetarian and vegan populations, caution must be taken when interpreting their results in research and clinical settings.

In the meantime, it remains important for clinicians to gain an in-depth understanding of veg*an patients reasons around food exclusion and dietary rules to ensure their eating habits are not being over-pathologized for simply following a veg*an diet. Furthermore, clinicians should also enquire about when they began their diet adherence to determine where exactly the diet fits into the patient’s history of disordered eating (i.e., did veg*ism predate the onset of the eating disorder or did veg*ism start as a result of the eating disorder) [41]. While weight restoration is best achieved through the reintroduction of meat [80], doing so in veg*ans who attribute their diet to reasons unrelated to weight loss or health (e.g., ethical, environmental, religion) may be detrimental to their treatment as a whole [1, 81]. Questions about motivations for veg*ism and sequence of events should be re-asked throughout treatment as patients may initially minimise the extent to which their veg*an diet is weight control or health motivated and the role it plays in the development and/or maintenance of their eating disorder [41]. As veg*ism is also known to be highly intertwined with ones sense of identity [6, 7], clinicians should focus on developing dietary flexibility in the context of a vegetarian or vegan diet to ensure the patients sense of self is maintained during treatment, and subsequent recovery [1, 41]. However, patients will gain greater insight into these concepts as treatment progresses.

### Future research

Research must establish whether commonly used eating disorder scales are accurately quantifying pathological eating behaviours in veg*ans. Based on the findings of this review, we recommend future research conduct an exploratory factor analysis of these scales in veg*ans to assess whether a different factor structure provides a more appropriate fit. We also recommend future research provides support for construct validity by comparing test scores using a semi–structured interview such as the widely supported EDE [75]. If, through confirming the psychometric properties of commonly used eating disorders scales, they continue to perform poorly, this will demonstrate the need to develop novel eating disorder scales or subscales suitable for vegetarians and vegans. Once such tools have been validated in these populations, longitudinal research must be conducted to track eating behaviours and attitudes in individuals as they transition to a vegetarian or vegan diet. In doing so, researchers would be able to establish a potential causal or bidirectional relationship between veg*ism and disordered eating, in turn guiding evidence-based treatment approaches for these populations. Without longitudinal research, it remains unclear whether veg*ism increases the risk of developing an eating disorder, or vice versa.

### Conclusion

This study presents the first systematic review to examine the association between vegetarianism, veganism, and disordered eating. Our findings demonstrate a lack of agreement whether veg*ism is associated with greater rates of global disordered eating but does potentially demonstrate an association with greater rates of orthorexia nervosa. It is clear there are additional factors influencing the association between global disordered eating, vegetarianism, and veganism which may be related to the use of eating disorder scales that are not psychometrically fit for these populations. Understanding the psychometric properties of eating disorder scales in veg*ans is vital to ensure such measures are accurately measuring eating psychopathology. In the meantime, caution must be taken when interpreting eating disorder results in both research and clinical settings to ensure veg*ans are not being unfairly pathologized for their dietary adherence.

### What is already known on this subject?

Veg*ism may act as a socially acceptable way to restrict food intake and camouflage disordered eating. Some ED scales may be capturing normal veg*an-motivated food choices and behaviours.

### What does this study add?

We found a positive relationship between veg*ism and orthorexia nervosa, but no consensus with global disordered eating. There is not enough evidence to support the use of ED scales in veg*ans.

## Data Availability

Data sharing is not applicable as no new data were created or analysed in this study.
